# Advanced Pancreatic Cancer Patient Benefit From Personalized Neoantigen Nanovaccine Based Immunotherapy: A Case Report

**DOI:** 10.3389/fimmu.2022.799026

**Published:** 2022-02-22

**Authors:** Jie Shao, Qin Liu, Jie Shen, Xiaoping Qian, Jing Yan, Yahui Zhu, Xin Qiu, Changchang Lu, Lanqi Cen, Manman Tian, Juan Du, Baorui Liu

**Affiliations:** The Comprehensive Cancer Center of Drum Tower Hospital, Medical School of Nanjing University & Clinical Cancer Institute of Nanjing University, Nanjing, China

**Keywords:** pancreatic cancer, neoantigen nanovaccine, immunotherapy, T-cell responses, benefit

## Abstract

Personal neoantigen vaccines are considered to be effective methods for inducing, amplifying and diversifying antitumor T cell responses. We recently conducted a clinical study that combined neoantigen nanovaccine with anti-PD-1 antibody. Here, we reported a case with a clear beneficial outcome from this treatment. We established a process that includes comprehensive identification of individual mutations, computational prediction of new epitopes, and design and manufacture of unique nanovaccines for this patient. Nanovaccine started after a relapse in third-line treatment. We assessed the patient’s clinical outcome and circulating immune response. In this advanced pancreatic cancer patient, the OS associated with the vaccine treatment was 10.5 months. A peptide-specific T-cell response against 9 of the 12 vaccine peptides could be detected sequentially. Robust neoantigen-specific T cell responses were also detected by IFN-γ ELISPOT and intracellular cytokine staining. In conclusion, sustained functional neoantigen-specific T cell therapy combined with immune checkpoint targeting may be well suited to help control progressive metastatic pancreatic cancer.

## Introduction

Pancreatic cancer is one of the most common cancers in the world. It is a devastating malignant disease with a median survival of 3-6 months and a 5-year survival rate of less than 5% (1, 2). As in most other countries, the health burden of pancreatic cancer is increasing in China, where the annual death rate is almost equal to the incidence (3). Pancreatic cancer is forecasted to become the second leading cause of cancer-related deaths by 2030 (4). Surgery is the best hope for curing pancreatic cancer, but only 20% of patients can relocate the tumor by the time they are diagnosed. Current therapies are severely lacking; recently approved combination chemotherapies such as FOLFIRINOX and gemcitabine/nab-paclitaxel only improve median survival by only 2–4 months and are associated with significant adverse effects (5, 6). Although some long-term survivors are beginning to be observed after such treatment, the 5-year survival rate is still grim at 8% (7).

In recent years, immunotherapy plays an increasingly important role in the treatment of solid tumors (8–11). Immune checkpoint pathway inhibitors show high response rates and persistent responses in melanoma, lung cancer, and kidney cancer. Currently, many clinical trials seek to evaluate the effectiveness of immunotherapy strategies for pancreatic cancer, including adoptive cell transfer, immune checkpoint inhibitors, cancer vaccines, and combinations with other immunotherapeutic agents. Blockade of immune checkpoints by anti-PD-1/anti-PD-L1 and/or anti-CTLA-4 agents lead to T cell activation and provide an effective approach for tumor immunotherapy. However, despite showing promising results in some malignancies, most Phase I and II clinical trials have failed to show any clinical efficacy in pancreatic cancer (8, 12). Immune checkpoints in combination with other approaches may be effective for pancreatic cancer.

Cancer vaccines have shown an advantage in treating solid tumors. Individual cancer neoantigen vaccines are capable of eliciting a powerful T-cell response and have been shown to achieve significant clinical efficacy (13, 14). Several recent phase I clinical trials have provided support for the hypothesis and have heralded a nascent era of personalized vaccines in the field of immunotherapy (15–17). The vaccines used to treat pancreatic cancer are varied and employ very different mechanisms. In addition to GVAX whole cell vaccine, only a few antigens have been evaluated as candidate vaccines for pancreatic cancer immunotherapy. Most of these antigens have been analyzed in combination with standard chemotherapy or immune checkpoint inhibitors. These vaccines have been shown to be safe, but of limited effectiveness (18–20). The limited efficacy of existing vaccine candidates in cases of pancreatic cancer with poor immunogenicity underscores the need to identify effective neoantigens for immunotherapy.

Advances in the definition of neoantigens provide a wealth of options for cancer treatment. Another advance in vaccine development is engineered nanoparticles (NPs), which serve as a vaccine delivery platform to protect the antigenic components of the vaccine while delivering innovative adjuvants that fine-tune the immune response (21–23). Nocardia rubra cell‐wall skeleton (N‐CWS) is an immunotherapeutic agent for cancer that has been shown to have the ability to activate an immune response without showing toxicity. There is evidence that N-CWS can activate macrophages and induce killer T cells (24). N‐CWS can be used as immunostimulatory therapy for DC proliferation and phenotypic and functional maturation (25).

In a Phase Ib clinical trial, Ott et al. demonstrated the feasibility, safety, and immunogenicity of a combination of personalized neoantigen vaccine and PD-1 inhibition in the treatment of advanced solid tumors. Vaccine-induced T cells persist for a period of time, exhibit cytotoxicity, and can metastasize to tumors. Epitope spread and major pathological tumor reactions were detected after vaccination (26). Based on the above principles, neoantigen nano vaccines combined with immune checkpoint inhibitors are considered to be a promising approach for the treatment of advanced cancer. Therefore, we conducted a clinical study on the combination of the neoantigen nano vaccine with anti-PD-1 antibody based integration of immunotherapy. Here, we report a case with a clear beneficial outcome from this treatment. Publication of any potentially identifiable images or data contained in this article requires written informed consent of the individual.

## Case Presentation

**Figure 1:**
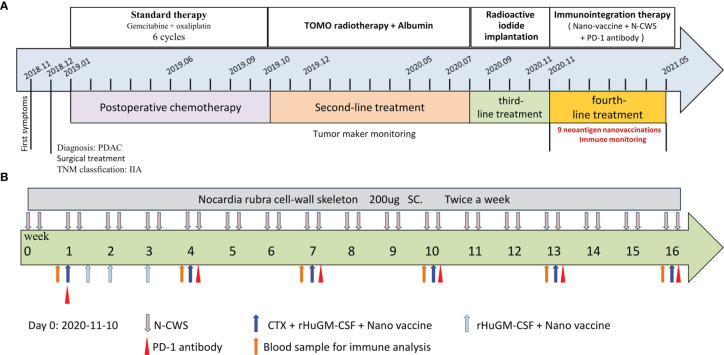
Process of clinical therapy. **(A)** The patient received different treatments at different times. Firstly, gemcitabine combined with oxaliplatin based postoperative chemotherapy (2019-01 to 2019-10, SD). Secondly, tomotherapy combined with albumin based second-line treatment (2019-10 to 2020-08, SD). Thirdly, radioactive iodide implantation was performed (2020-08 to 2020-11, PD). Finally, neoantigen nanovaccine combined with immune checkpoint inhibitors based immunotherapy. **(B)** The course of immunotherapy. The patient received nine doses of the neoantigen nanovaccine and also received intravenous injection of PD-1 antibody and subcutaneous injection N-CWS during course of treatment.

**Figure 2:**
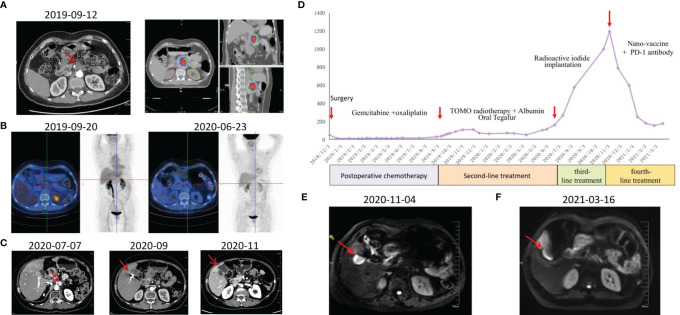
Examinations of patient in different periods. **(A)** CT scans revealed retroperitoneal lymph node metastasis 9 months after surgery and tomotherapy was performed. **(B)** PET-CT scans were performed before and approximately 9 months after treatment. **(C)** CT scans during third-line treatment. **(D)** CA19-9 levels of this patient throughout the treatment. **(E)** MRI scan was performed before immunotherapy. **(F)** MRI scan was performed approximately 4.5 months after immunotherapy.

The patient was female, 67-year-old. Radical resection of body and tail of pancreas was performed after the diagnosis of PDAC in Dec-2018. According to UICC TNM nomenclature the tumor was classified as a completely resected, poorly differentiated ductal adenocarcinoma stage IIA, pT3pN0M0. After surgery, the patient received 6 cycles of gemcitabine plus oxaliplatin. Unfortunately, the enhanced CT scan in Sep-2019 revealed retroperitoneal lymph node metastases. The patient received second line treatment: tomotherapy plus albumin paclitaxel. The cumulative dose for retroperitoneal metastatic lymph nodes was: Planning Target Volume (PTV): 50Gy/10f, as shown in **Figure 2A**. After second line treatment, the positron emission tomography-computed tomography (PET-CT) scans showed retroperitoneal metastatic lymph nodes became smaller, though the metabolism is similar to what it was before, as shown in **Figure 2B**. From July 2020 to September 2020, the patient underwent two radioactive iodide implants, during which time the tumor markers CA19-9 continued to rise and new lesions appeared in the liver, and the patient was comprehensively evaluated for PD, as shown in **Figure 2C**. Time line of events is detailed in **Figure 1A**.

Because of the continued rise of CA-199 and the emergence of new lesions, the patient herself strongly requested to be administrated to our clinical trial of personalized neoantigen nanovaccine combined with anti-PD-1 antibody based immunotherapy. According to the patient gene expression profile, substrings within the 15 mers that had a binding affinity of less than 500 nM for this patient’s HLA allele were considered as candidates and synthesized Methods for predicting and identifying antigenic peptides were provided in **Supplementary Material**. Top 12 predicted binding peptides restricted by autologous MHC class I and class II allotypes were synthesized (**Table 1**).

**Table 1:** HLA-binding peptides for patient.

NO.	Gene	Mutation amino acid	HLA type	No. of peptide	Sequence of neoantigen	Mutant peptide Affinity (nM)	VAF (%)
1	LRRC37A3	p.R1313H	HLA-B*4001	9	RSHMTHRTPK	16.3	2.5
2	PRKAG2	p.P220L	HLA-A*1101	10	ASLTHYAPSK	17.99	2.9
3	DDX11	p.V246M	HLA-B*3901	9	MKSLGSVQL	35.56	1.5
4	PRPF8	p.R510H	HLA-A*1101	9	MLNLLIHHK	43.4	3.5
5	ALS2	p.I1639R	HLA-A*4001	10	GEQGRMFTTL	49.55	8.7
6	SPAG9	p.P587L	HLA-A*1101	10	TSHVTLSVKK	55.48	2.6
7	BCLAFI	p.N627S	HLA-A*1101	10	LSERFTSYQK	89.6	3.9
8	KCNMAI	p.K711X	HLA-B*4001	9	MEACGTHPT	457.84	6.6
9	PARP4	p.V458I	HLA-DRB1*1501	15	NIVGILCRGLLLPKI	186.97	8.6
10	SIGLEC10	p.Q144K	HLA-DRB1*1101	15	GFFLKVTALTKKTVR	23.96	7.6
11	KRAS	p. G12v	HLA-A*0201	10	KLVVVGAVGV	156.45	1.9
12	ATM	p.S2168L	HLA-A*0201	9	RSLESVYLL	219.53	1.7

To preparation of neoantigen nanovaccine, we introduced a cysteine residue to the C-terminal of the neoantigen peptide. The free sulfhydryl group provided the potential to connect peptide to the DSPE-PEG-Mal through Michael addition reaction (**Supplementary Material**). The patient received a 500 μg dose per peptide of nanovaccine by subcutaneous injection on day 1, Day 3, Day 7, Day 14, Day 21, Day 42, Day 63, Day 84, Day 105 respectively (**Figure 1B**), along with adjuvant chemotherapy in the form of Montanide ISA 51 VG and 100 μg GM-CSF. Throughout the duration of vaccine treatment, the patient received subcutaneous injections of N‐CWS twice a week. Only transient fever and local rash occurred during nanovaccine treatment. As shown in **Figure 2D**, CA19-9 declined rapidly with the use of the nanovaccine-based immunotherapy. Fortunately, the Magnetic Resonance Imaging (MRI) scan performed 4.5 months after immunotherapy showed a remarkable regression of liver lesion as shown in **Figures 2E, F**. Eventually, the patient died in September 2021, and the OS associated with the nanovaccine treatment was 10.5 months.

**Figure 3:**
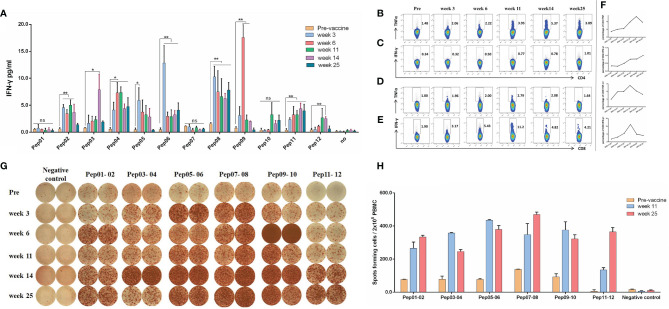
Immune responses of neoantigen nanovaccine. **(A)** Cytometric bead array assays demonstrated IFN-γ secretion by PBMCs before and in different periods after immunotherapy following overnight culture with neoantigen peptides and control. **(B)** The percentage of TNF-α-positive cells among CD4+ T cells before and in different periods after neoantigen nanovaccine following 12-day culture with peptide pool. **(C)** The percentage of IFN-γ-positive cells among CD4+ T cells. **(D)** The percentage of TNF-α-positive cells among CD8+ T cells. **(E)** The percentage of IFN-γ-positive cells among CD8+ T cells. **(F)** Line Graph. **(G)** IFN-γ ELISPOT showed changes in peptide-specific IFN-γ secretion by patient PBMCs before and in different periods after immunotherapy following 12-day culture with neoantigen peptides. We stimulated PBMCs with a mixture of two antigenic peptides, pre-stimulated PBMCs (2x105per well) with irradiated autogenous PBMC loaded with mixed peptide were added to duplicate wells for 18–20 hours. For example, antigen peptide 01 and antigen peptide 02 were combined to form a peptide pool, antigen peptide 03 and antigen peptide 04 were combined to form a peptide pool, and so on. **(H)** Histogram of FN-γ ELISPOT assay. The Graphpad Prism 5.0 software was used for all statistical analysis. p-values < 0.05 were significant, as indicated with asterisks. (*p < 0.05; **p < 0.01; ns, not significant).

To confirm the immunogenicity of candidate neoantigen peptides for this patient at a series of time points pre- and post-vaccination, peripheral blood was collected from the patient prior to immunization (Week 0), and again at Weeks 3, 6, 11, 14 and 25 post-vaccination (**Supplementary Material**). The immunogenicity of each of the 12 mutations peptide administered in this study was analyzed by IFN-γ release measured by cytometric bead array after overnight co-culture of PBMCs that were pulsed with the indicated neoantigen peptide. As shown in **Figure 3A**, Judging from the secretion level of IFN-γ in the culture supernatant, the patient’s response to 9 out of 12 peptides after each cycle of injection showed a dynamic change, which were statistically significant. With the exception of pep01, pep07 and pep10, the secretion of IFN-γ by other peptide-stimulated T cells was significantly increased compared with that before nanovaccine treatment.

For ICS, the peptide pool was a mixture of 12 peptides. As shown in **Figure 3B**, the percentage of TNF-α-producing CD4+ T cells kept going up until week 14 (1.48% vs 5.37% for Pre vs week14). As shown in **Figure 3C**, although the percentage of IFN-γ-producing CD4+ T cells was relatively low, but it kept rising. Meanwhile, the percentage of TNF-α-producing CD8+ T cells kept going up until week 11(1.00% vs 2.79% for Pre vs week11), so did the percentage of IFN-γ-producing CD8+ T cells, It peaked at week 11, which was 5.8 times higher than before treatment, as shown in **Figures 3D** and **3E**. The line graph of Intracellular cytokines is shown in **Figure 3F**.

The results of in vitro IFN-γ Elispot showed that the immune response of the patient’s antigen-specific T lymphocytes to these peptides exhibiting dynamic changes over time, AS shown in **Figure 3G**. For Elispot assay, we stimulated PBMCs with a mixture of two antigenic peptides, pre-stimulated PBMCs (2×10^5^per well) with irradiated autogenous PBMC loaded with mixed peptide were added to duplicate wells for 18–20 hours. For example, antigen peptide 01 and antigen peptide 02 were combined to form a peptide pool, antigen peptide 03 and antigen peptide 04 were combined to form a peptide pool, and so on. Because the number of cells tested was not enough, we only did duplicate wells. AS shown in **Figure 3H**, To be defined as a vaccine-induced response, the frequency of cytokine-positive cells within one combinatorial pattern had to be at least twofold over the frequency in the corresponding mock control and the frequency before vaccination. As shown in **Figure 3H**, the frequency of IFN-γ-positive cells at week 11 increased by three or four times over the frequency before vaccination.

## Discussion

This is a case of immune integration therapy with obvious benefits. The patient progressed after multi-line therapy and then received individualized nanovaccine-based immune integration therapy. Fortunately, the patient benefited from nanovaccine-based immunotherapy. The OS associated with the nanovaccine treatment was 10.5 months. The treatment was effective and had few side effects, so the patient was satisfied with the treatment.

Despite the therapeutic effect of immune checkpoint blockade, most solid tumor patients treated with anti-PD-1/PD-L1 monotherapy did not achieve objective response, and most tumor regression was partial rather than complete in pancreatic cancer (27–29). It is speculated that the lack of preexisting anti-tumor immunity and/or the presence of additional tumor immunosuppressive factors in the tumor microenvironment are responsible for such treatment failures. Cancer vaccines can prepare for immune checkpoint blockade therapy by inducing effective anti-tumor immunity, especially in patients lacking tumor-infiltrating T cells (26, 30). We hypothesize that effective vaccination in pancreatic cancer patients along with interventions that can reprogram important immunosuppressive factors in the tumor microenvironment can enhance tumor immune recognition, thus enhancing response to PD-1/PD-L1 blockade.

Conventional tumor therapeutic vaccines usually select tumor-associated antigen that are highly expressed by tumor cells, but showed limited therapeutic efficacy. Neoepitopes derived from somatic mutation appear to represent ideal targets for T vaccine–based cancer immunotherapy. Our preclinical animal studies demonstrated the number of tumor-infiltrating T cells nanovaccine treatment group was significantly higher than free vaccine treatment group, and the article is in the submission.

This is a case of comprehensive treatment with obvious benefits. In the first stage, the patient benefited from postoperative chemotherapy and second-line treatment, and the disease stabilized for some time. But serum CA19-9 started to increase and CT scans showed new metastatic lesions in the liver, so the patient herself requested to be administrated to our clinical trial of personalized neoantigen nanovaccine combined with anti-PD-1 antibody based immunotherapy. After 9 cycles of vaccine treatment, CA19-9 declined rapidly and the Magnetic Resonance Imaging (MRI) scan performed 3.5 months after immunotherapy showed a remarkable regression of liver lesion and this patient had significantly beneficial results from neoantigen nanovaccine combined with immune checkpoint inhibitors.

The immunogenicity of each of the 12 mutation peptides administered in this study was analyzed by IFN-γ release, 9 out of 12 peptides after each cycle of injection showed a dynamic change. The results of *in vitro* IFN-γ Elispot showed that the immune response of the patient’s antigen-specific T lymphocytes to these peptides exhibiting dynamic changes over time. In view of the importance of CD8+ and CD4+ T cells in mediating tumor cell killing, the nanovaccine, by utilizing MHC-I peptides and MHC-II peptides, was designed to activate both types of T cells. A substantial proportion of antigen-peptide-specific CD4+ and CD8+ T cells were detected. Our detection methods are limited, and we will use more methods to prove the effectiveness of nanovaccines in future studies. For example, tetramer detection, single cell transcriptome analysis, neoantigen-specific TCR clonotypes diversify, epitope spreading of T cell responses and gene expression profiles in individual neoantigen reactive T cell.

In conclusion, we demonstrate that the T-cell response could be produced by the personal neoantigen nanovaccine in patients with advanced pancreatic cancer. The combination of functional neoantigen-specific T cells with immune checkpoint targeting therapy may help control progressive advanced pancreatic cancer. Larger randomized clinical trials are needed to identify and validate the possibility of designing a nano-vaccine for pancreatic cancer in future.

## Data Availability Statement

The original contributions presented in the study are included in the article/**Supplementary Material**. Further inquiries can be directed to the corresponding authors.

## Ethics Statement

The studies involving human participants were reviewed and approved by Medical Ethics Committee of Drum Tower Hospital Affiliated to Nanjing University Medical School. The patients/participants provided their written informed consent to participate in this study. Written informed consent was obtained from the individual(s) for the publication of any potentially identifiable images or data included in this article.

## Author Contributions

QL and BL designed the clinical trial. JSha and JD composed the manuscript and provided figures. YZ, XQ, MT, XQ, LC and CL did the work of the acquisition, analysis and interpretation of the data. JShe and JY revised the manuscript critically for important intellectual content, and agreement to be accountable for all aspects of the work, in ensuring that questions related to the accuracy or integrity of any part of the work are appropriately investigated and resolved. All authors contributed to the article and approved the submitted version.

## Funding

This work was supported by grants from the National Natural Science Foundation of China (NO. 81902914).

## Conflict of Interest

The authors declare that the research was conducted in the absence of any commercial or financial relationships that could be construed as a potential conflict of interest.

## Publisher’s Note

All claims expressed in this article are solely those of the authors and do not necessarily represent those of their affiliated organizations, or those of the publisher, the editors and the reviewers. Any product that may be evaluated in this article, or claim that may be made by its manufacturer, is not guaranteed or endorsed by the publisher.
