# Supplementary material for: Advanced Pancreatic Cancer Patient Benefit From Personalized Neoantigen Nanovaccine Based Immunotherapy: A Case Report
Source: Front Immunol. 2022 Feb 22;13:799026. doi: 10.3389/fimmu.2022.799026 (PMC8901600; doi:10.3389/fimmu.2022.799026)
Supplement: Supplementary file 1 [file DataSheet_1.docx]

**Supplemental Information**

**Advanced Pancreatic Cancer Patient Benefit From** **Personalized Neoantigen Nanovaccine Based Immunotherapy: A Case Report**

Jie shao*, Qin Liu*, Jie Shen, Xiaoping Qian, Jing Yan, Yahui zhu, Xin Qiu, Changchang Lu, Lanqi Cen, , Manman Tian, Juan Du^#^ and Baorui Liu^#^

The Comprehensive Cancer Center of Drum Tower Hospital, Medical School of Nanjing University & Clinical Cancer Institute of Nanjing University, Nanjing 210008, China

*These authors contributed equally to this work

Correspondence and requests for materials should be addressed to
J.D. (email: dujuanglyy@163.com ) or B.L. (email:baoruiliu@nju.edu.cn)

Methods

**Prediction and identification of target epitopes**

Patient-specific neoantigens were selected on the basis of tumor-specific mutations identified by RNA-seq. RNA was extracted from unstained FFPE sections using the miRNeasy FFPE Kit (cat# 217504, Qiagen) according to the manufacturer’s protocol. The yield and quality of extracted RNAs were assessed using the Qubit RNA HS Assay Kit (Thermo Fisher Scientific) and LabChip GX Touch HT Nucleic Acid Analyzer (PerkinElmer), respectively. After ribosomal RNA depletion by the NEBNext rRNA Depletion Kit (cat# E6310L, New England Biolabs), cDNA synthesis was performed with M-MLV RT RNase (H-) (cat# M3683, Promega) and NEB Second strand mRNA synthesis kit (cat#E6111L, New England Biolabs). Sample library preparation was performed using the KAPA Hyper Prep Kit (KAPA Biosystems) and subjected to sequencing on a NovaSeq 6000 platform with 2×151 bp paired-end reads according to the manufacturer’s instructions. The relative abundance of each annotated transcript was reported as transcripts-per-million (TPM) and log2-transformed before analysis. Sequencing was performed and analyzed by OrigiMed.

OptiType was used to identify class I human leukocyte antigen (HLA-A, HLA-B, and HLA-C) alleles and class Ⅱ HLA-DRB1 allele. After all nonsynonymous mutations were identified by NGS, the mutant epitopes were predicted by NetMHCpan v3.0 and NetMHCII 2.2 tools as we described previously (ref?). Substrings within the 15 mers that had a binding affinity of less than 500 nM or % rank < 2.0 for any patient’s HLA allele were considered as candidates and synthesized. Top 12 predicted binding peptides restricted by autologous MHC class I and class II allotypes were synthesized (table 1). The synthesis of peptide should meet the following criteria, including bacteria-free, >95.0% purity, good solubility, quantities of bacterial endotoxin less than 10 EU/mg.

**Preparation and characterization of nanovaccines**

2-Distearoyl-sn-glycero-3-phosphoethanolamine-N-[hydroxysuccinimidyl (polyethylene glycol)-2000] (DSPE-PEG2000-NHS) and mix peptides at a molar ratio of 1:1.5 were mixed in PBS. The mixture was agitated overnight with a magnetic stirrer at room temperature. Finally, the amphiphilic polymers were purified through dialysis, lyophilized, and stored at -80°C for further use. Nanovaccine size and zeta potential were measured using a dynamic light scattering analyzer (Malvern Instruments Corporation, United Kingdom). To assess its stability, samples of prepared nanovaccine were pre-incubated with PBS for 120 hours at 4°C and 37°C. Electron microscopy was performed using an electron microscope equipped with a CCD camera and operating at 80 kemV. Dynamic light scattering analysis revealed that the particle size of nanoparticles peaked at 20–30 nM and it was slightly negatively charged. The size and polydispersity index of nanovaccine remained stable for up to 96 hours at either 4℃ or 37℃

**I****mmune monitoring**

**Patient samples**

All the experimental methods were carried out in accordance with the approved guidelines. The blood collection procedure was carried out in accordance with the guidelines verified and approved by the Ethics Committee of Drum Tower Hospital. The patient signed an informed consent for scientific research statement. Patient peripheral blood mononuclear cells (PBMCs) were isolated by Ficoll/Hypaque density-gradient centrifugation (GE healthcare) and cryopreserved with 10% dimethyl sulfoxide in FBS (Sigma-Aldrich). Cells from patients were stored in vapor-phase liquid nitrogen until the time of analysis.

**Analysis of T cell responses**

PBMCs were thawed and rested overnight in AIM-V medium (Gibco) supplemented with 10% FCS (Gibco). For ex vivo analysis with the IFN-γ Flex Set, 2×10^5^ PBMCs were plated in triplicate in 96-well cell culture plates with individual peptides (25µg/ml)) and incubated overnight and supernatant was harvested for further testing. Recognition of the single antigens was tested as compared with no-peptide (media) control, and stimulus phytohemagglutinin was used as positive control. For in vitro expansion (‘pre-stimulation’) of antigen-specific T cells, IL-7 (25 ng/ml; PeproTech) and ow-dose IL-2 (20 U/ml) were added to culture medium. Supplementation of cytokines and half-medium change were performed every 3 d, as described previously. After 11 days of stimulation, cells were used in ELISpot assays or intracellular cytokine staining.

Cytometric bead array analysis of cytokines

The concentrations of cytokines in culture supernatants were measured by cytometric bead array according to the manufacturer’s protocol (BD Biosciences) with an appropriate diluent. Human IFN-γ Flex Set (Bead B8) (BD Biosciences) was used for detection of single-cytokine IFN-γ. The samples were run and FACS data were collected using an Accuri C6 (BD Biosciences) flow cytometer and analyzed using FCAP version 3.0 array software (Soft Flow).

IFN-γ ELISPOT assay

For pre-stimulated PBMCs, IFN-γ ELISPOT kit (Dakewei) was used to determine the frequency of cytokine-secreting T cells after overnight activation with irradiated autogenous PBMC loaded with peptide. Briefly, pre-stimulated PBMCs (2x105per well) with irradiated autogenous PBMC loaded with peptide were added to duplicate wells for 18–20 hours. The plates were washed before the addition of the diluted detection antibody (1:100 dilutions) and then incubated for 1 hour in 37°C. After washing of the plates, streptavidin–HRP (1:100 dilutions) was added and incubated at 37°C for another 1 hour. 3-Amino-9-ethylcarbazole (AEC) solution mix was then added to each well, and the plates were left in the dark for about 15–25 minutes at room temperature before deionized water was added to stop development. Plates were scanned by ELISPOT CTL Reader (Cellular Technology Inc.), and the results were analyzed with Elispot software (AID). Spots greater than twice the no-peptide (media) control were considered positive for T cell reactivity.

**Intracellular cytokine staining**

After 11 days of stimulation, ICS was performed and 1× 10^6^ antigenic peptides stimulated PBMC per well were re-stimulated with irradiated autogenous PBMC at an effector to target ratio of 2:1, pooled peptides (each at 1.5–2μg ml-1) were added to the culture. Stimulated T cells were treated with GolgiStop (BD Biosciences) according to the manufacturer’s recommendations for 8h the following day. After treatment, T cells were stained for 30 min at room temperature with a fixable live/dead stain (FVS780), anti-CD3 (BUV395), anti-CD8 (PerCP-Cy™5.5) and anti-CD4 (FITC). Cells were fixed and permeabilized (Fixation/Permeabilization Solution Kit, BD Biosciences). Intracellular cytokines were stained with anti IFN-γ (PE) and TNF-α (APC) (BD Biosciences) for 1 h at 4 °C. Cells were washed with permeabilization buffer and fixed (1% paraformaldehyde solution, Sigma-Aldrich). Cells were analyzed using a CytoFLEX.
